# Understanding the Meaning of a Good Death for People Living With Parkinson's Disease: Qualitative Study

**DOI:** 10.1111/jgs.70541

**Published:** 2026-06-30

**Authors:** Leonardo Martins, Rasa Mikelyte, Ricardo Silva Carvalho, Henrique Ballalai Ferraz, Déborah Oliveira, Julia Maria Vanelli, Natália Rocha Tardelli, Fernanda Bono Fukushima, Edison Iglesias de Oliveira Vidal

**Affiliations:** ^1^ Public Health Department Medical School Botucatu, São Paulo State University (UNESP) Botucatu Brazil; ^2^ Centre for Health Services Studies, University of Kent Canterbury UK; ^3^ Internal Medicine Department Medical School Botucatu, São Paulo State University (UNESP) Botucatu Brazil; ^4^ Movement Disorders Division, Neurology Department Federal University of São Paulo São Paulo Brazil; ^5^ Universidad Andrés Bello Faculty of Nursing Viña del Mar Chile; ^6^ Millenium Institute for Care Research (MICARE) Santiago Chile; ^7^ Geriatrics Division, Internal Medicine Department Medical School Botucatu, São Paulo State University (UNESP) Botucatu Brazil; ^8^ Pain and Palliative Care Division, Surgical Specialties and Anesthesiology Department Medical School Botucatu, São Paulo State University (UNESP) Botucatu Brazil

**Keywords:** death, palliative care, Parkinson's disease, qualitative studies

## Abstract

**Background:**

Parkinson's disease is the second most common neurodegenerative disorder globally. Despite growing attention to palliative care in Parkinson's disease, little is known about what constitutes a “good death” from the perspective of people living with Parkinson's disease (PLwPD).

**Objective:**

To explore the meaning of a good death for PLwPD.

**Methods:**

In this cross‐sectional multicentre qualitative study, we conducted semi‐structured interviews with 30 PLwPD recruited through purposive sampling from four geriatric and neurology outpatient clinics between May 2021 and December 2022. Transcripts were analyzed using inductive thematic analysis. The process involved independent coding and iterative discussions grounded in a constructionist paradigm.

**Results:**

The sample was diverse in terms of race, gender, age, religious affiliation, educational background, and disease stage. We identified two major themes related to the participants' last days of life: Fears and Coping. Reported fears included experiencing disability, pain and discomfort, fear of feeling shame, fear of being a burden, fear of being abandoned and left helpless. Coping was a multidimensional theme, comprising the relational experience of feeling well cared for (defined by being valued, receiving clear and honest communication, and being treated with love and kindness) alongside the active strategies of finding opportunities for joy and drawing on religiosity and spirituality. Religiosity/spirituality appeared as a key factor in emotional regulation, fostering a sense of purpose and acceptance in the face of death.

**Conclusion:**

Our findings suggest that improving palliative care for PLwPD requires an approach that actively addresses specific fears and strengthens the multiple dimensions of coping, which include fostering opportunities for joy, supporting spirituality, and enhancing the relational experience of feeling well cared for. This study illuminates often‐overlooked aspects of care and provides a basis for the development of person‐centered interventions aimed at enhancing the quality of dying—and of life—in this population.

## Summary

- Key Points
  ○ People living with Parkinson's articulate fears about disability, pain, shame, and abandonment when contemplating end of life.
  ○ Coping dimensions include feeling well cared for, finding joy, and drawing on religiosity and spirituality for emotional regulation and purpose.
  ○ Achieving a good death involves tipping the balance by strengthening coping mechanisms and reducing fears.
- Why Does This Paper Matter?
  ○ This study explored the concept of a good death from the perspective of People Living with Parkinson's Disease (PLwPD). While healthcare professionals often focus primarily on the motor and non‐motor symptoms of Parkinson's, this study's key contribution lies in deepening our understanding and sensitivity to several overlooked fears and dimensions of coping that are relevant for PLwPD and play important roles in the shaping of experiences in their last days of life.

## 1 Introduction

Parkinson's disease (PD) is the second most common neurodegenerative disorder affecting older adults and has been proposed as an ideal model condition for chronic neurological disorders, under the assumption that improvements in the care for People Living with PD (PLwPD) could be applied to other chronic neurologic disorders [1, 2]. Despite its growing impact, various distressing symptoms, such as pain, depression, and constipation, remain underassessed and undertreated [3, 4]. Additionally, psychosocial, spiritual, practical, and carer burden‐related concerns are often underestimated. Notably, the provision of palliative care to PLwPD is often delayed or entirely absent [1, 5]. PD is also associated with an increased risk of developing dementia, a condition that introduces significant challenges to end‐of‐life care due to the progressive loss of patient autonomy in decision‐making [6, 7]. Recently, the need for greater investment in palliative care for this population has gained recognition, with documented benefits including enhanced quality of life, improved symptom management, and strengthened support for carers [8, 9].

The planning of end‐of‐life care and the development of new models of care for PLwPD reflect a growing commitment to improving the overall quality of care for this population. Several studies have explored the concept of a “good death” from the patients' perspective across different conditions [10, 11, 12, 13]. Despite increasing recognition of the importance of palliative care in PD, little is known about what constitutes a good death from the perspective of PLwPD. Given the importance of such understanding for providing appropriate palliative care, this study aimed to explore this concept through a qualitative approach.

## 2 Methods

This study employed a cross‐sectional qualitative design grounded in the constructionist paradigm, which posits that reality is socially constructed and shaped by individuals' beliefs, practices, and interactions within society [14]. Inductive thematic analysis was chosen as the analytical method due to its theoretical flexibility and suitability for exploring the perspectives of individuals [15].

All participants had their decision‐making capacity to provide informed consent assessed concurrently with the presentation of the Informed Consent Form, following the guidelines proposed by Sudore [16].

The study was approved by the Research Ethics Committees of the Botucatu Medical School (CAAE: 36453420.1.0000.5411) and the Paulista School of Medicine (CAAE: 36453420.1.3001.5505). In preparing this report, we followed the Consolidated criteria for Reporting Qualitative research (COREQ) guidelines [17].

### 2.1 Sample

We used purposive sampling aimed at achieving maximum diversity in age, race, educational level/social class, religious affiliation, and stage of PD among participants from four health services (two specialized movement disorders clinics, a tertiary geriatric clinic, and a primary care geriatric clinic) in two different cities in the state of São Paulo, Brazil: Botucatu and São Paulo. Participants were initially invited in person by healthcare professionals working in those services. When potential participants agreed to have their contact information shared with the research team, they were contacted by phone to receive further information and an invitation to participate. The sample size was determined based on the concept of Information Power, a model used to guide sample adequacy in qualitative studies considering factors such as the study's aim, sample specificity, theoretical framework, quality of dialog, and analytical strategy [18]. Participant recruitment was discontinued once the data collected were sufficiently rich and diverse to yield nuanced insights and adequately address the study's objectives without the need for additional data to enhance meaning or interpretation.

### 2.2 The Interview Procedure

Interviews were conducted between May 2021 and December 2022, using a semi‐structured topic guide (File S1) developed by the research team based on a previous study [19]. The interview guide was pilot tested with one PLwPD before the start of the study. Depending on logistical considerations, such as participant preference and geographic distance, interviews were conducted either in person at participants' homes (audio‐recorded) or remotely via the Google Meet platform (audio‐ and video‐recorded). Field notes were made after the interviews when contextual or interactional features not fully captured by the recordings were considered relevant. Interviews were conducted by three researchers trained in qualitative methods not hitherto known to the participants: A male physical therapist (LM) with extensive experience working with PLwPD, a female (JMV) and a male (RSC) medical students, both with limited experience working with PLwPD.

Following the interviews, sociodemographic data were verbally collected, including age, self‐reported gender and race, years of education, approximate time since diagnosis, religious affiliation, relationship with the primary carer, and whether the carer cohabited with the participant. Data on PD staging were obtained using the Hoehn & Yahr classification system, based on participant responses regarding mobility, balance, disability, and the presence of unilateral or bilateral parkinsonian symptoms [20]. Participants were accordingly classified from Stage 1 (unilateral involvement with minimal or no functional disability) to Stage 5 (confined to bed or wheelchair unless aided).

### 2.3 Data Analysis

The content of the interviews was transcribed verbatim and analyzed using inductive thematic analysis, which involves identifying, analyzing, and reporting recurring patterns across the data. The process followed six stages: (1) familiarization with the data, (2) generation of initial codes, (3) identification of themes, (4) review of themes, (5) definition and refinement of thematic narratives, and (6) production of the final report [21]. We used the Taguette software during the coding process of the thematic analysis [22]. Three researchers (LM, RSC, and EIOV) coded the data independently, then met to discuss, refine, and standardize coding approaches.

### 2.4 Reflexivity

Our research team was composed of professionals from diverse clinical and academic backgrounds, including physical therapy, nursing, medicine (geriatrics, palliative care, pain medicine, and neurology), and psychology, as well as two medical students. We recognized that this dominant clinical positionality inclined us toward a “problem‐solving” lens and deficit‐oriented interpretations.

Differences in prior experience working with PLwPD were intentionally leveraged during analysis. In particular, the physical therapist's clinical experience sensitized the team to functional and embodied dimensions of participants' narratives, especially those related to disability and loss of independence. In contrast, team members with more limited prior exposure to PLwPD contributed a stance of curiosity that helped preserve participants' language and meanings, reducing the risk of premature clinical framing.

Through iterative discussions, these perspectives were brought into dialog, with researchers explicitly challenging one another's assumptions. This reflexive engagement supported more nuanced interpretations while maintaining analytic grounding in participants' accounts.

## 3 Results

We conducted 30 interviews, 13 of which were held in participants' homes, while the remaining 17 were conducted online. Three people who initially had shown interest in the study later declined to participate or did not respond to follow‐up contacts. The median interview duration was 27 min, with a range from 17 to 164 min. All but eight interviews had a carer or family member present based on the participants' preferences or needs. All participants were interviewed a single time.

Twenty‐two (73%) participants were aged ≥ 60 years; almost two‐thirds were male, and around one sixth were Black (Table 1). Most participants were Christians and in Stages 3 and 4 of PD, in which there are progressive activity limitations while individuals remain ambulatory [23].

**TABLE 1:** Characteristics of participants.

Characteristic	*N* (%) or median (range)
Age, median (range)	67 (36–84)
Gender, *N* (%)	
Female	11 (37%)
Male	19 (63%)
Race, *N* (%)	
Black	5 (17%)
White	25 (83%)
Years of education, median (range)	8 (1–20)
Time since diagnosis in years, median (range)	7 (3–40)
Stage of Parkinson's Disease^a^, *N* (%)	
Stage 1	5 (17%)
Stage 2	5 (17%)
Stage 3	10 (33%)
Stage 4	9 (30%)
Stage 5	1 (3%)
Relationship with primary carer, *N* (%)	
Spouse	2 (7%)
Daughter	1 (3%)
Son	4 (13%)
Daughter‐in‐law	1 (3%)
Friend	1 (3%)
Does not need a carer	21 (70%)
Carer lives with participant	7 (23%)
Religious affiliation, *N* (%)	
Catholicism	18 (60%)
Evangelicalism	9 (30%)
Spiritism	2 (7%)
Buddhism	1 (3%)

^a^ Hoehn & Yahr staging of Parkinson's Disease ranges from 1 to 5, with 1 representing unilateral symptoms only and minimal disability, and 5 for people that are wheelchair‐bound or bedridden unless aided.

We identified two major themes from the data, reflecting complementary dimensions of the concept: Fears and Coping. Each theme encompassed several subthemes, which captured specific fears and dimensions of coping (Figure 1).

**FIGURE 1:**
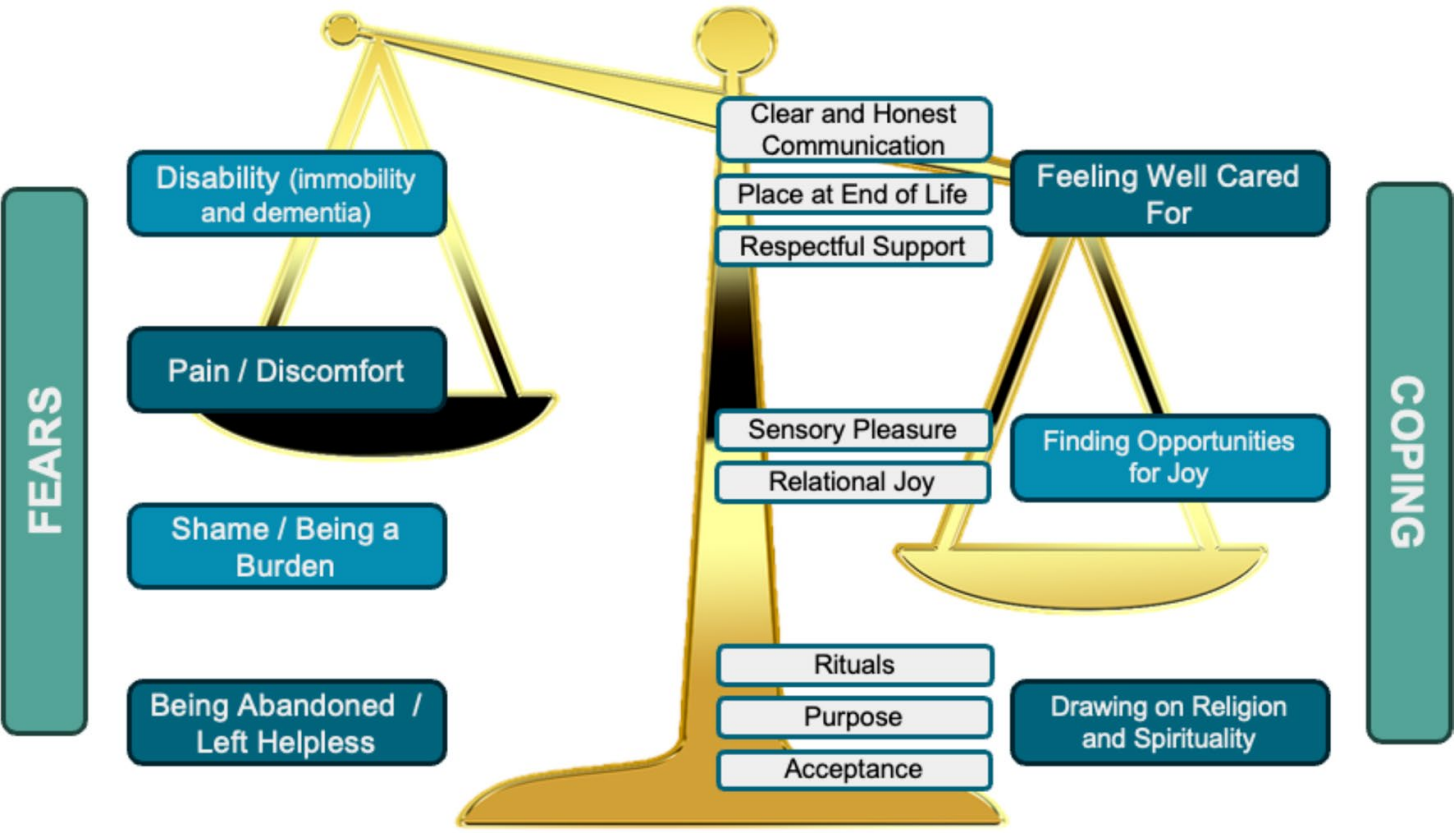
Thematic model derived from qualitative interviews with people living with Parkinson's disease, showing two main themes related to their last days of life: Fears and Coping. The balance‐scale metaphor represents the dynamic and ongoing negotiation between fears and coping resources, rather than the elimination or cancelation of fear.

### 3.1 Fears

Fear played a central role in how participants articulated their views on their last days of life. It manifested across a range of dimensions, encompassing both physical and psychosocial concerns. These included experiences related to their own disease trajectory, observations of others living with PD or facing end‐of‐life situations, as well as imagined scenarios. Based on thematic analysis, these fears were grouped into four subthemes: (1) fear of disability; (2) fear of pain and discomfort; (3) fear of shame and of being a burden; and (4) fear of abandonment and helplessness.

#### 3.1.1 The Fear of Disability

Participants often expressed fears related to the anticipated progression of their disease and the consequent loss of independence. Imagining a future marked by continuous struggle often provoked anxiety. The prospect of losing occupational, social, motor, mental, and self‐care abilities was perceived as a profound threat to one's identity and dignity as an autonomous individual (Table 2, quotes 1–3).

**TABLE 2:** Select quotes for the fears theme organized by subtheme.

Quote #	Selected quotes from participants on the fears theme
The fear of disability	
1	*“From what I've seen… I think… I'm going to… be, be bedridden… needing… to stay… with almost no movement at all, because I'm losing my mobility, I'm noticing… and needing someone to… turn me to the side in bed, to sit me up, to feed me…*”*(P11)*
2	*“Being stuck in a bed… not being able to move, not being able to do anything… I think it's a huge amount of suffering… I'm scared of that.”* *(P17)*
3	*“Dying stuck in bed, unable to move, it's a dirty trick!” (P26)*
4	*“…about the way I die… you don't need… I don't need to wait until I'm completely debilitated, right? I can die before I get to that point […] It's just that we… we just watch, you know? Like spectators watching the story… and I find that so awful, just… in such bad taste! Yeah… watching the decline, watching it go on, and on, and on… and then suddenly, it ends in a really bad way, with so much suffering…* [*…*] *Ugh… suffering in relation to everything… the limitations we have to face… your body not obeying you, your body just… suffering… all the stuff that comes with… with the illness […] I find it unfortunate to live in a country that doesn't have euthanasia. Oh, euthanasia is so necessary, isn't it? I've talked to… to some people during my life, in Europe, with people who said, “You know, in your country I don't know, but in mine… there are many doctors who decide to end a life, be it a child or an adult, whatever… when they realize there's no way out, they end it.” (P14)*
5	*“I don't want it, I've already said, if I get to the point where I see that things are getting ugly, ah, I'm not gonna let it go any further*(*…*), *look, honestly, to be real, yeah, I'd take my own life. If I see that I'm gonna become dependent on everything, then that'll be it!” (P08)*
The fear of shame and being a burden	
6	*“I think like this, being in bed depending on others, just for someone to clean me up, that's the only thing I don't agree with… I could die right away without going through this situation… I just don't want my husband to come and clean my butt… That's my concern, it's embarrassing… it just… it bothers me only in that aspect… depending on others, for me… as for the rest, I'll manage, take it in stride.” (P04)*
7	*“If… I end up bedridden… and need someone to go to the bathroom, or can't get up anymore, having to wear, uh, diapers… then that's tough…* [*…*] *My my mother passed away too… and she asked me to take her to the toilet… and even though there were people, in the family, women, at home, I still had to take her… her doing her business… I said ‘mom, this is so embarrassing!’, I said that… having to do that… ‘embarrassing for us, for me and for you’.” (P23)*
8	*“the patient, from the day that he… starts depending on… on other people to help… In my opinion, right there life is over! You get me? Someone taking you to the bathroom, washing you, giving you a bath, putting food in your mouth… Pushing the person to eat… So, I mean, the guy is living… for the other person, not for himself!” (P24)*
9	*“No, I'm not afraid… not even of death, I'm not afraid! [My fear] is this, being in a bed, suffering there, causing worry for those in the house, and… and I wouldn't want to have… I would want, if it were my request, a quick death!” (P13)*
10	*“[To die] without anyone seeing, right? To die and, when you look, you're already resting there! … The best thing! Because then you don't suffer much, right? Can you imagine? You're dying and the person beside you? Can you imagine them seeing you in your final moments there? It's agonizing, isn't it?” (P29)*
11	*“If I'm causing trouble and you guys aren't able to give me that support, I won't be sad, put me in a nursing home!” (P02)*
The fear of pain and discomfort	
12	*“I guess pain is the toughest part, isn't it? If we have pain… I think the most important thing is pain, right? What I'm afraid of, doctor, is suffering! [laughs] I have a lot of pain sometimes… Now, if a person becomes bedridden, then… Being bedridden, I think, I don't know, it's very tough, isn't it? It's not easy, no!” (P06)*
13	*“It must be really awful to die from not being able to breathe! My dad has asthma, you know, and I've seen him have really bad attacks where he couldn't breathe… It's really terrible, right? The person is begging for help, you know, asking for help with their eyes, right?” (P08)*
14	*“The agony of death must be terrible… like the person is drowning, I don't know… Yeah, pain… but it's more the part of the, the, the… shortness of breath, for example, all those things…” (P03)*
15	*“I think death is painful! And I… I'm a little afraid of death! […] death is painful from what I've seen!” (P11)*
The fear of being abandoned and left helpless	
16	*“I do have a fear, a fear of progressing and ending up without, without… without anyone to take care of me… like if I end up in a wheelchair all the time, becoming helpless in bed like that…” (P03)*
17	*“I just want my relatives to understand this… and… from the moment it starts to, you know, tremble, if I ever get there… right?… that people understand that this is not, it's not a, it's not a procedure of the, of the, of the… disease, (…) so… The hard part is… people who end up like this, and the family simply abandons them… right? So, a person who worked their whole life… right… when they become someone useless, let's put it that way, not a useless person, but… not fit for anything, not even for their… for their own thoughts… People abandon them and just let it go… that's terrible, isn't it?” (P07)*
18	*“[When] you get to the end of life at the time when you need energy, happiness, affection the most, and you lose it all… my other son… he won't have the patience, he won't have time to look after me” (P25)*
19	*“I think that… because of my family, they won't leave me… throw me in a… how do you say? Like, in a nursing home and forget about me, right?” (P24)*
20	*“I'm afraid of… of being alone [in my last hours]!” (P27)*

Confronted with fears regarding disease progression and the resulting loss of functionality and independence, one participant expressed a desire for euthanasia, while another contemplated suicide as a means of preempting future suffering. Interestingly, when euthanasia was mentioned, it was framed within a paternalistic context, wherein the responsibility for such a decision was attributed to the physician rather than the patient (Table 2, quotes 4–5).

#### 3.1.2 The Fear of Shame and Being a Burden

Another facet of the fears expressed by participants involved how they perceived their disease progression through the eyes of others. For example, some participants expressed fears regarding the prospect of becoming unable to perform their personal hygiene autonomously and eventually needing a carer or family member to assist them. This resulted in a complex mix of fear, embarrassment, and shame. Such extreme dependence was perceived as the ultimate stage of autonomy loss, where modesty and dignity could no longer be preserved (Table 2, quotes 6–8).

Beyond the dependence on care, concern for the well‐being of loved ones often involved fears related to their emotional suffering. Frequently, the wish for a swift death or to be alone at the time of death was primarily motivated by a desire to spare family members from both emotional distress and the burden of caregiving (Table 2, quotes 9–11).

#### 3.1.3 The Fear of Pain and Discomfort

Participants feared the occurrence of several symptoms at the end of life. Some of these fears were based on personal experiences with PD, such as pain, while others were stemmed from witnessing the illness of others, such as shortness of breath (Table 2, quotes 12–15).

#### 3.1.4 The Fear of Being Abandoned and Left Helpless

In the anticipated context of progressive loss of autonomy and increasing burden to carers, some participants expressed fears of being abandoned by their family members. This imagined abandonment was associated with a state of extreme vulnerability and accompanied by a sense of uselessness. The suffering linked to this prospect of abandonment manifested on two levels: one rooted in a feeling of injustice, and the other in a perceived loss of self‐worth (Table 2, quotes 16–20).

### 3.2 Coping

Almost as a mirror image of participants' fears, we derived a multidimensional coping theme, comprising the relational experience of feeling well cared for alongside the active strategies of finding opportunities for joy and drawing on religiosity and spirituality.

#### 3.2.1 Feeling Well Cared for

“Feeling well cared for” synthesized various elements through which participants perceived their fears could be alleviated. It involved three subthemes related to the place of death, the characteristics of the care provided by health and care professionals, and the relationship with their families.

Most participants expressed a preference for dying at home, as this setting embodied the symbolism of home, that is, a space associated of safety, values, and affection. However, dying in a hospital environment emerged as an acceptable alternative in the face of the possibility of better symptom control, thereby minimizing suffering through appropriate therapies and ensuring comfort and dignity in a critical context (Table 3, quotes 1–2).

**TABLE 3:** Select quotes for the coping theme organized by subtheme.

Quote #	Select quotes from participants on the coping theme
Feeling well cared for	
1	*“Ah, [I'd rather die] in my sleep, right?! And not wake up anymore… In our own home, right? Yeah, you sleep and wake up dead. Because it's the best place in our home, right? There may be nothing there, but there's no place like home!” (P04)*
2	*“Yeah, I'd like to die in a hospital!… Oh, suddenly you're in a hospital, you're being taken care of, but you're not going to make it, you know?… or maybe, so I don't have so much suffering.” (P28)*
3	*“Oh, [healthcare professionals can help] by embracing the patient in both their ethical and professional roles, with a lot of love?” (P06)*
4	*“They [the healthcare professionals] must tell the truth to the person… this is what they have to do! […] Sometimes they know that the person will not make it, then it will prepare the person, right?” (P25)*
5	*“I think that… he [the doctor] should, he… would have to give the patient all the… um… the guidance that the patient needed. He wouldn't just leave the patient to their own devices… I give the medicine, and that's it—it's not like that! I think he has to have more, more awareness of what he's doing in relation to, to, to the person… the person who chose him as a doctor deserves a little more attention… and respect…” (P15)*
6	*“I wish there were someone… The children… I would die more… peacefully, right?… I don't know if (death will be) peaceful… for me… [their presence] is a demonstration… of love, right?” (P11)*
7	*“I don't really want anything in particular [for my last hours of life]. Not even having family around or anything, ‘cause… you know, watching someone die isn't easy. It's a heavy thing, especially when it's someone you love. Makes it even worse. So yeah…” (P07)*
Finding opportunities for joy	
8	*“I think, oh, don't let me run out of my cigarettes, and don't let me run out of my sweets! Chocolate!! Chocolate, especially! [It would be] ideal!” (P08)*
9	*“I really love to travel, you have no idea! To keep traveling. If I could die, if I could die while traveling. Yeah, the way I like it! It's a wish, because I love to travel!” (P04)*
10	*“[Speaking of a friend who was dying] She said she wanted to eat a croquette. [The carer] said she had just finished eating, and she was joyful, as if nothing had happened, as if she were going on a beautiful trip… She ate, and it was like she really wanted to eat, hungry, I don't know, and it was such a joy for her…” (P20)*
11	*“That the whole family would come together, but I don't know if it's possible. Before I die, right? I wanted to see everything like that, well, right? […], my wish was that, you know? I always think about that… Then I would leave very happy.” (P17)*
12	*“In my last moments I pray every day for my family to all have their health, joy, and to be by my side! … seeing everyone sitting there at the table having lunch, for me that's the greatest satisfaction I have! My kids, my grandkids, my whole family! […]”[Family being there brings] Everything, joy, health, even health! Even if it's in my last moments!” (P01)*
13	*“[I'd like my family] to sing the songs I like most… on the trips I used to take with my kids we were always singing songs… It's a joy to be near them, it's a joy to be around them.” (P16)*
Drawing on religiosity and spirituality	
14	*“No. No, I just thought, on the day of my death, that my wife or anyone else (…) with my nephew… to play Ave Maria by Gounod.” (P07)*
15	*“God shows up everywhere and He's always right there with us! So He, He gives us strength!” (P05)*
16	*“As a Catholic that I am, I, I, I would like to, I really want to, go through a confession, with a priest, right? Receive the sacrament of communion and be prepared to go to God, right? Confession leads me to have forgiveness for my sins…” (P30)*
17	*“Yeah, I'm no better than anyone else, serving God the way I'm serving, right? I'm like this, not totally prepared, but yeah… Happy with the situation I live with Parkinson's! Because I serve God, and I can't fear this part […] that's what's important, and to have a calm, peaceful death, without fear […] If He wants to take me away! [laughs] I'm already going happy! […] For me, for me it's a good way! For me it is!”(P02)*
18	*“Well, in the end, nobody can do anything!… Nobody can do anything! Just have…’it's… as they say,’it's… pray to God that everything goes well… look out for those who stay… and t'at's it!” (P13)*
19	*“Religion helps me a lot… if I have the light, if God shows me the way, I have everything! Even with sickness, I have everything!” (P26)*
20	*“[Healthcare professionals] seem to have that gift that God gave them! (…) So what they talk with people… Not all of them, but they are pleasant with the person at these times… at least the ones I know, have always been like that.” (P17)*

Health and care professionals played an important role in comprehensive care. Participants expressed a desire for support, attention, respect, honest communication, and, interestingly, affection, combined with continuity of care, as factors that would foster a sense of security (Table 3, quotes 3–5).

A quality relationship with families was considered a central component of a good death, as participants believe that fear and suffering could be mitigated through love and connection. Importantly, for some participants, there was also a tension between the desire for closeness with family and the wish to protect them from the distress of witnessing their death (Table 3, quotes 6–7).

#### 3.2.2 Finding Opportunities for Joy

Some participants expressed a desire for opportunities to experience joy in the face of their final moments of life. These wishes took various forms, ranging from sensory and gustatory experiences to engaging in specific activities such as traveling, pursuing hobbies, and even shopping. A common feature across these desires was the shift in focus from dying to living, achieved through identifying potential sources of joy (Table 3, quotes 8–10).

In addition to the wishes for sensory sources of joy mentioned above, participants also expressed a desire for relational joy, which arises through interactions with loved ones. Beyond the previously noted reassuring and alleviating role of family during the last moments of life, interviewees emphasized the presence of family members as a source of happiness at the end of life, perhaps reflecting a longing to relive, even briefly, the emotional warmth and joy experienced in earlier shared moments, such as family gatherings during celebrations (Table 3, quotes 11–13).

#### 3.2.3 Drawing on Religiosity and Spirituality

The theme of religiosity and spirituality encompassed desires for practices that foster some form of spiritual dialogue, such as the anointing of the sick and confession with religious representatives. These practices were regarded as highly meaningful, serving as mechanisms for emotional regulation by addressing fears related to suffering and uncertainty. They represented pathways to spiritual comfort, strength to face adversity and the transition to death, as well as a sense of clear conscience, for example, through the forgiveness of sins (Table 3, quotes 14–16).

Religiosity and spirituality also conveyed to participants a sense of purpose and acceptance. Focusing on achieving goals related to their religious beliefs provided a feeling of agency while offering a pathway to peace. At the same time, religious convictions often helped participants come to terms with aspects related to their illness and future death that lay beyond their control. Acknowledging that the circumstances of their deaths were inexorable and determined by God's will, which, according to their religious views, is inherently good, promoted acceptance and alleviated fears. Importantly, prayer also appeared to promote emotional regulation, while reinforcing a sense of agency and acceptance in the face of death (Table 3, quotes 17–18).

Religiosity and spirituality also served as interpretive lenses through which participants made sense of their experiences, enhancing the comfort derived from them. For example, health and care professionals were often perceived as bearers of a divine gift, manifested through their actions and conversations aimed at bringing comfort to those under their care (Table 3, quotes 19–20).

## 4 Discussion

This study explores the concept of a good death from the perspective of PLwPD. Our findings align with prior literature on the concept of a good death in other clinical contexts, while also offering novel insights that are particularly relevant for the provision of palliative care for PLwPD.

Our findings on the concept of a good death for PLwPD can be illustrated by the image of a scale (Figure 1): on one side, the many fears they experience; on the other, the resources and strategies they draw upon to cope with and alleviate those fears. Importantly, this image is not meant to suggest that coping mechanisms eliminate or cancel fears, but rather to represent an ongoing and often fragile negotiation between coexisting vulnerabilities and supports. From this perspective, dying well involves tipping the balance by strengthening coping mechanisms and reducing fears. A key contribution of this study lies in deepening our understanding and sensitivity to these fears and dimensions of coping. Healthcare professionals caring for PLwPD often focus primarily on issues related to the motor and non‐motor symptoms of that illness while other dimensions remain overlooked. Our results have the potential to enhance clinicians' awareness of patients' fears and coping strategies that may help mitigate them. For instance, some of the fears reported by our participants, such as embarrassment and shame at the prospect of a spouse performing intimate hygiene, fear of being abandoned as someone “useless” or “without value”, and fear of being a source of suffering for loved ones, are rarely identified or addressed in clinical practice.

Several previous studies have explored the concept of a good death across different patient populations. A systematic review identified key elements shaping this concept among individuals with serious illnesses and older adults [24]. The identified core elements were: effective pain and symptom management; being recognized and treated as a person; clear communication and shared decision‐making; the ability to contribute to others; a sense of closure; and preparation for death. The main themes influencing these elements included religion and spirituality, cultural and societal factors, financial concerns, life circumstances, age, and disease.

On the one hand, several elements from our findings align with those reported in previous reviews, as, for example, the fear of pain and discomfort, the wish for honest communication with healthcare professionals, the fear and shame of becoming a burden to loved ones, and the importance of religiosity and spirituality. On the other hand, our study offers additional insights specific to individuals with PD that were not addressed in those reviews. In particular, the fear of disability was a central theme for our participants with PD yet was absent from the prior literature on a good death. This suggests that concerns about losing mobility and independence are distinctive factors shaping how people with PD think about their end of life, and which require careful attention from healthcare professionals. In contrast to people with cancer, whose diagnosis is often equated with dying [23], PD is more strongly linked to the notion of disability, which then becomes a central fear when contemplating the end of life.

Another important insight that appeared in our study but was absent from previous reviews on a good death concerns the value of attempting to create opportunities for joy in the last days of life. This topic was not addressed in sections devoted to palliative care within influential guidelines on the general management of PD [25, 26], nor in guidelines specifically focused on palliative care for PLwPD [5, 9]. Interestingly, it was also absent from previous studies on the needs of PLwPD and what matters most to them [27, 28, 29]. Indeed, a recent review concluded that joy among older adults has received little attention in the gerontological literature, particularly in the healthcare context [30].

The value in identifying and promoting opportunities for joy in the face of serious illnesses has been recognized by several health professions including geriatricians, neurologists, palliative care, and mental health professionals [31, 32, 33, 34]. Although the growing age‐friendly health system movement lists questions such as “what brings you joy?” under the “What Matters” component of the 4Ms framework, the implementation of that domain often remains insufficiently integrated into clinical care [35, 36, 37, 38]. Our study serves as a reminder to clinicians caring for people with PD of the significance of asking about and attempting to promote joy even in the last days of life.

Our study has several limitations. First, we were only able to interview PLwPD who felt comfortable discussing issues related to death and dying. As a result, the perspectives of individuals who were unwilling to engage with this topic are likely underrepresented in our findings, a limitation inherent to research on sensitive end‐of‐life topics. Second, the study was conducted exclusively with patients residing in two cities within a single state in one Latin American country. As is typical in qualitative research, our goal was not to produce statistical generalizations but to deepen understanding of what constitutes a ‘good death’ for PLwPD, with the understanding that it is a profoundly culturally‐rooted concept that may not be transferable to other settings. Importantly, in qualitative studies, transferability is understood as a collaborative process between researchers and readers [39, 40]. Based on the researchers' descriptions and interpretations, readers assess the extent to which the findings resonate with and are applicable to other contexts that share similar characteristics. Fourth, we did not return transcripts to participants for comment or conduct member checking [41]. Although increasingly used in qualitative interview‐based studies, its contribution to credibility remains debated, and the process may pose emotional or cognitive burdens for participants [42, 43].

Despite these limitations, our findings have implications for clinical practice across geriatric, neurological, and palliative care settings. Rather than proposing new goals for neuropalliative care, they offer empirical, patient‐derived specificity that can help operationalize the identification and addressing of patients' needs in concrete, actionable clinical encounters (Table S1). In the context of a chronic, progressive neurological condition such as Parkinson's disease, the fears and coping resources articulated by participants illuminate not only end‐of‐life experiences but also the broader quality of care across the disease trajectory, underscoring the close interrelationship between living well and dying well [13]. Participants described distinct and sometimes underrecognized concerns (e.g., fear of progressive disability, shame related to intimate dependency, fear of abandonment) alongside coping resources (e.g., relational care, joyful activities, and spiritual/existential supports) that can serve as a practical map for anticipatory guidance, communication, and interdisciplinary care planning throughout the course of illness.

## 5 Conclusion

Our findings suggest that improving palliative care for PLwPD requires an approach that actively addresses specific fears and strengthens the multiple dimensions of coping, which include fostering opportunities for joy, supporting spirituality, and enhancing the relational experience of feeling well cared for. This study illuminates often‐overlooked aspects of care and provides a basis for the development of person‐centered interventions aimed at enhancing the quality of dying—and of life—in this population.

## Author Contributions

L.M. and E.I.O.V. had full access to all the data in the study and take responsibility for the integrity of the data and the accuracy of the data analysis and are the guarantors for the study. Concept and design: L.M. and E.I.O.V. Acquisition of data: L.M., R.S.C., and J.M.V. Analysis: L.M., R.S.C., R.M., J.M.V., D.O., H.B.F., N.R.T., F.B.F., and E.I.O.V. Drafting of the manuscript: L.M. Critical revision of the manuscript for important intellectual content: R.S.C., R.M., J.M.V., D.O., H.B.F., N.R.T., F.B.F., and E.I.O.V.

## Funding

This study was funded in part by the São Paulo Research Foundation (FAPESP)—Brazil (process number #2021/09729‐0), the Brazilian National Council for Scientific and Technological Development (CNPq)—Brazil (process number 312499/2022‐1), and the Coordenação de Aperfeiçoamento de Pessoal de Nível Superior—Brazil (CAPES) (process number #8888.7.502948/2020‐00).

## Disclosure

The funders of this study played no role in the design, methods, subject recruitment, data collections, analysis and preparation of paper. A preprint version of this manuscript is available on medRxiv (https://doi.org/10.1101/2025.11.17.25340426).

## Conflicts of Interest

The authors declare no conflicts of interest.

## Linked Article

This publication is linked to an editorial by Li and Harrison. To view this article, visit https://doi.org/10.1111/jgs.70604.

## Supporting information


**Table S1:** Translating participant‐derived themes into practical prompts and interdisciplinary actions in the care of People Living with Parkinson's Disease.

## Data Availability

Because we do not have the permission of participants to share the transcribed interviews and to preserve their anonymity, the transcribed interviews are not available for sharing. In case other researchers wish to have access to the anonymized interview transcripts, they should contact the corresponding author while understanding that any access to the transcripts is conditional to the approval by the specific Ethics Review Committee.

## References

[1] B. R. Bloem , E. J. Henderson , E. R. Dorsey , et al., “Integrated and Patient‐Centred Management of Parkinson's Disease: A Network Model for Reshaping Chronic Neurological Care,” Lancet Neurology 19, no. 7 (2020): 623–634, 10.1016/S1474-4422(20)30064-8. PMC9671491 32464101

[2] D. Su , Y. Cui , C. He , et al., “Projections for Prevalence of Parkinson's Disease and Its Driving Factors in 195 Countries and Territories to 2050: Modelling Study of Global Burden of Disease Study 2021,” BMJ 388 (2025): e080952, 10.1136/bmj-2024-080952. PMC11881235 40044233

[3] J. M. Miyasaki , “Palliative Care in Parkinson's Disease,” Current Neurology and Neuroscience Reports 13, no. 8 (2013): 367, 10.1007/s11910-013-0367-y. 23761129

[4] K. E. Lyons and R. Pahwa , “The Impact and Management of Nonmotor Symptoms of Parkinson's Disease,” American Journal of Managed Care 12, no. S17 (2011): S308–S314. 22087551

[5] S. Fox , A. Cashell , W. G. Kernohan , et al., “Palliative Care for Parkinson's Disease: Patient and Carer's Perspectives Explored Through Qualitative Interview,” Palliative Medicine 31, no. 7 (2017): 634–641, 10.1177/0269216316669922. 27683476

[6] M. A. Hely , W. G. J. Reid , M. A. Adena , G. M. Halliday , and J. G. L. Morris , “The Sydney Multicenter Study of Parkinson's Disease: The Inevitability of Dementia at 20 Years,” Movement Disorders 23, no. 6 (2008): 837–844, 10.1002/mds.21956. 18307261

[7] C. H. Williams‐Gray , S. L. Mason , J. R. Evans , et al., “The CamPaIGN Study of Parkinson's Disease: 10‐Year Outlook in an Incident Population‐Based Cohort,” Journal of Neurology, Neurosurgery, and Psychiatry 84, no. 11 (2013): 1258–1264, 10.1136/jnnp-2013-305277. 23781007

[8] S. Timmons and S. Fox , Palliative Care in People With Parkinson's Disease: Guidelines for Professional Healthcare Workers on the Assessment and Management of Palliative Care Needs in Parkinson's Disease and Related Parkinsonian Syndromes (University College Cork, 2016), https://hospicefoundation.ie/wp‐content/uploads/2021/01/Palliative‐Care‐in‐people‐with‐Parkinsons‐disease.pdf.

[9] L. Greensall and H. Currie , “Clinical Guideline for the Management of Patients With Parkinson's Disease Who are Approaching the end of Life,” (2024), https://www.westmidspallcare.co.uk/wp‐content/uploads/Parkinsons‐Guideline‐SPAGG‐v4‐08.08.24.pdf.

[10] E. A. Meier , J. V. Gallegos , L. P. M. Thomas , C. A. Depp , S. A. Irwin , and D. V. Jeste , “Defining a Good Death (Successful Dying): Literature Review and a Call for Research and Public Dialogue,” American Journal of Geriatric Psychiatry 24, no. 4 (2016): 261–271, 10.1016/j.jagp.2016.01.135. PMC4828197 26976293

[11] K. E. Steinhauser , E. C. Clipp , M. McNeilly , N. A. Christakis , L. M. McIntyre , and J. A. Tulsky , “In Search of a Good Death: Observations of Patients, Families, and Providers,” Annals of Internal Medicine 132, no. 10 (2000): 825–832, 10.7326/0003-4819-132-10-200005160-00011. 10819707

[12] S. Payne , A. Langley‐Evans , and R. Hillier , “Perceptions of a “Good” Death: A Comparative Study of the Views of Hospice Staff and Patients,” Palliative Medicine 10, no. 4 (1996): 307–312, 10.1177/026921639601000406. 8931066

[13] R. H. Harwood , J. A. Thiyagarajan , A. Bhadelia , et al., “Measuring and Monitoring the Quality of Dying in the UN Decade of Healthy Ageing,” Lancet Healthy Longevity 6, no. 7 (2025): 100739, 10.1016/j.lanhl.2025.100739. 40716442

[14] C. E. Rees , P. E. S. Crampton , and L. V. Monrouxe , “Re‐Visioning Academic Medicine Through a Constructionist Lens,” Journal of the Association of American Medical Colleges 95, no. 6 (2020): 846–850, 10.1097/ACM.0000000000003109. 31809294

[15] M. E. Kiger and L. Varpio , “Thematic Analysis of Qualitative Data: AMEE Guide No. 131,” Medical Teacher 42, no. 8 (2020): 846–854, 10.1080/0142159X.2020.1755030. 32356468

[16] R. L. Sudore , C. S. Landefeld , B. A. Williams , D. E. Barnes , K. Lindquist , and D. Schillinger , “Use of a Modified Informed Consent Process Among Vulnerable Patients: A Descriptive Study,” Journal of General Internal Medicine 21, no. 8 (2006): 867–873, 10.1111/j.1525-1497.2006.00535.x. PMC1831581 16881949

[17] A. Tong , P. Sainsbury , and J. Craig , “Consolidated Criteria for Reporting Qualitative Research (COREQ): A 32‐Item Checklist for Interviews and Focus Groups,” International Journal for Quality in Health Care 19, no. 6 (2007): 349–357, 10.1093/intqhc/mzm042. 17872937

[18] K. Malterud , V. D. Siersma , and A. D. Guassora , “Sample Size in Qualitative Interview Studies: Guided by Information Power,” Qualitative Health Research 26, no. 13 (2016): 1753–1760, 10.1177/1049732315617444. 26613970

[19] R. Mikelyte , K. H. Dening , D. Oliveira , et al., ““It's My Life, It's My Choice and I Want to Say When” vs “A Good Death Is to Be on Good Terms With God”. Comparing the Views of People With Dementia in the UK and Brazil About a Good Death: A Cross‐Cultural Qualitative Study,” BMC Palliative Care 24, no. 1 (2025): 138, 10.1186/s12904-025-01771-w. PMC12082953 40380168

[20] M. M. Hoehn and M. D. Yahr , “Parkinsonism: Onset, Progression, and Mortality,” Neurology 17, no. 5 (1967): 427, 10.1212/WNL.17.5.427. 6067254

[21] V. Braun and V. Clarke , “Using Thematic Analysis in Psychology,” Qualitative Research in Psychology 3, no. 2 (2006): 77–101, 10.1191/1478088706qp063oa.

[22] R. Rampin and V. Rampin , “Taguette: Open‐Source Qualitative Data Analysis,” Journal of Open Source Software 6, no. 68 (2021): 3522, 10.21105/joss.03522.

[23] R. P. Moser , J. Arndt , P. K. Han , E. A. Waters , M. Amsellem , and B. W. Hesse , “Perceptions of Cancer as a Death Sentence: Prevalence and Consequences,” Journal of Health Psychology 19, no. 12 (2014): 1518–1524, 10.1177/1359105313494924. PMC4099292 23864071

[24] A. Krikorian , C. Maldonado , and T. Pastrana , “Patient's Perspectives on the Notion of a Good Death: A Systematic Review of the Literature,” Journal of Pain and Symptom Management 59, no. 1 (2020): 152–164, 10.1016/j.jpainsymman.2019.07.033. 31404643

[25] D. Grimes , M. Fitzpatrick , J. Gordon , et al., “Canadian Guideline for Parkinson Disease,” Canadian Medical Association Journal 191, no. 36 (2019): E989–E1004, 10.1503/cmaj.181504. PMC6733687 31501181

[26] Parkinson's Disease in Adults: Diagnosis and Management (National Institute for Health and Care Excellence (NICE), 2017), http://www.ncbi.nlm.nih.gov/books/NBK447153/. 28787113

[27] S. Dorrance , R. Patel , G. T. Stebbins , et al., “It Is Personal: What People With Parkinson's Disease Say Matters Most for Quality of Life,” Movement Disorders 41, no. 3 (2026): 741–749, 10.1002/mds.70134. PMC13022584 41388603

[28] J. R. Mammen , M. Tyo , J. Cadorette , et al., “Understanding What Aspects of Parkinson's Disease Matter Most to Patients and Families,” Scientific Reports 14, no. 1 (2024): 21171, 10.1038/s41598-024-71555-4. PMC11387791 39256441

[29] F. P. Vlaanderen , L. Rompen , M. Munneke , M. Stoffer , B. R. Bloem , and M. J. Faber , “The Voice of the Parkinson Customer,” Journal of Parkinson's Disease 9, no. 1 (2019): 197–201, 10.3233/JPD-181431. 30373962

[30] V. A. Goodwin , A. J. Hall , A. Bethel , et al., “Understanding Joy Amongst Older People: A Scoping Review,” Archives of Gerontology and Geriatrics 115 (2023): 105118, 10.1016/j.archger.2023.105118. 37516061

[31] H. H. Bentz , S. H. Madsen , M. S. Pilegaard , et al., “Occupations Creating Joy for People Living With Advanced Cancer: A Qualitative Descriptive Study,” British Journal of Occupational Therapy 85, no. 3 (2022): 187–198, 10.1177/03080226211009419. PMC12033723 40337079

[32] B. M. Kluger and R. M. Arnold , “The Total Enjoyment of Life: A Framework for Exploring and Supporting the Positive in Palliative Care,” Journal of Palliative Medicine 26, no. 10 (2023): 1322–1326, 10.1089/jpm.2023.0321. PMC10541933 37471240

[33] A. D. Naik , L. A. Martin , J. Moye , and M. J. Karel , “Health Values and Treatment Goals of Older, Multimorbid Adults Facing Life‐Threatening Illness,” Journal of the American Geriatrics Society 64, no. 3 (2016): 625–631, 10.1111/jgs.14027. PMC5001155 27000335

[34] M. E. Tinetti , D. M. Costello , A. D. Naik , et al., “Outcome Goals and Health Care Preferences of Older Adults With Multiple Chronic Conditions,” JAMA Network Open 4, no. 3 (2021): e211271, 10.1001/jamanetworkopen.2021.1271. PMC7991967 33760091

[35] J. Adler‐Milstein , R. Thombley , S. Rosenthal , B. Rosner , and S. Rogers , “Assessing Equitable Adherence to the Age‐Friendly Health System's 4Ms Framework in an Academic Inpatient Setting,” INQUIRY: The Journal of Health Care Organization, Provision, and Financing 61 (2024): 00469580241285598, 10.1177/00469580241285598. PMC11425728 39314001

[36] S. Lesser , S. Zakharkin , C. Louie , M. R. Escobedo , J. Whyte , and T. Fulmer , “Clinician Knowledge and Behaviors Related to the 4ms Framework of age‐friendly Health Systems,” Journal of the American Geriatrics Society 70, no. 3 (2022): 789–800, 10.1111/jgs.17571. PMC9299469 34837381

[37] J. S. Powers and N. Penaranda , “Creation of a Whole Health Age‐Friendly Template and Dashboard Facilitates Implementation of 4Ms Into Primary Care,” Geriatrics 7, no. 5 (2022): 109, 10.3390/geriatrics7050109. PMC9601591 36286213

[38] M. E. Tinetti and B. S. Nettles , “What Matters About What Matters Most,” JAMA Network Open 8, no. 10 (2025): e2535775, 10.1001/jamanetworkopen.2025.35775. 41051780

[39] J. Ritchie , ed., Qualitative Research Practice: A Guide for Social Science Students and Researchers (Sage, 2014).

[40] R. E. Stalmeijer , M. E. L. Brown , and B. C. O'Brien , “How to Discuss Transferability of Qualitative Research in Health Professions Education,” Clinical Teacher 21, no. 6 (2024): e13762, 10.1111/tct.13762. 38497107

[41] C. McKim , “Meaningful Member‐Checking: A Structured Approach to Member—Checking,” American Journal of Qualitative Research 7, no. 2 (2023): 41–52.

[42] L. Birt , S. Scott , D. Cavers , C. Campbell , and F. Walter , “Member Checking: A Tool to Enhance Trustworthiness or Merely a Nod to Validation?,” Qualitative Health Research 26, no. 13 (2016): 1802–1811, 10.1177/1049732316654870. 27340178

[43] A. Erdmann and S. Potthoff , “Decision Criteria for the Ethically Reflected Choice of a Member Check Method in Qualitative Research: A Proposal for Discussion,” International Journal of Qualitative Methods 22 (2023): 16094069231177664, 10.1177/16094069231177664.

