# Supplementary material for: Understanding the Meaning of a Good Death for People Living With Parkinson's Disease: Qualitative Study
Source: J Am Geriatr Soc. 2026 Jun 30;74(10):3051–61. doi: 10.1111/jgs.70541 (PMC13723212; doi:10.1111/jgs.70541)
Supplement: Supplementary file 1 [file JGS-74-3051-s001.pdf]

Supplementary File S1

Guide for Semi-Structured Interview

Thank you for agreeing to take part in this interview. In this research, we would like to hear your opinion about what would constitute a good death.

This interview aims to improve our understanding of the needs and preferences of people with Parkinson's disease, with the goal of enhancing the quality of their end-of-life experiences.

If at any time during the interview you need to stop or leave the room, please let me know.

Everything that you tell me will be treated anonymously, avoiding your exposure.

At the end of the interview, if you have any additional comments or questions that you would like to share, please let me know, so I can record them.

The main goal of this interview is to understand:
- What a good death means to you
- What a bad death means to you
- What kinds of things could make a death "good"

QUESTIONS

1. First of all, what does a good death mean to you?

Potential prompts

- Do you remember having experienced the death of someone close to you in the past?
    - What was it like? Did the person die from a diagnosed illness? Do you know what it was?

2. Are there specific things that you would wish for your last days of life?

3. Are there specific things that you fear for your last days of life?

4. Does your Parkinson's diagnosis make any difference in what a good death means to you?

5. What do you understand about what might happen to you, as Parkinson's disease progresses?

6. Do you feel you know as much as you want to know about what may happen in your last days of life?

Potential prompts

- How important is it to you to know in advance what is likely to happen as you near death?
- What are your thoughts and feelings about the uncertainties around death and dying?
- What symptoms do you think people may experience when dying?
    - *Wait for spontaneous response. If the interviewee asks for examples, mention* pain, difficulty breathing, incontinence, inability to chew or swallow, or loss of consciousness.

7. Is it important for you to have control over your death? [If yes] What would having control feel like?

Potential prompts

- What kinds of preparations do you think can be made for a person's end of life?
- Have you made any preparations or thought about doing so? (For example, a will, an advance directive, etc.)

8. Would you like to be alone or have someone with you during your final days of life?

Potential prompts

- How important would it be to have family or friends nearby? How might the presence of family and friends influence the quality of your death?
- Would you like to have anyone else nearby, such as a healthcare professional, a representative of your religion (e.g., priest), or a pet?
- What kind of role would you like them to have?
- Is it important for you to be able to say goodbye to people? If so, when and how?

9. Where do you think would be a good place to die?

Potential prompts

- Do you have a preference for dying in your own home, someone else's home, or in a hospital? Why?
- Is there any place where you definitely would not want to die? Why?
- If dying in a hospital meant that your symptoms (for example, pain) would be better controlled, would that affect your choice of where to die and why?

10. How can health and social care professionals influence the quality of your death?

Potential prompts

- And what about nurses, doctors, and formal caregivers? Anyone else?

Supplementary Table S1: Translating participant-derived themes into practical prompts and interdisciplinary actions in the care of People Living with Parkinson's Disease.

What to listen for	Example prompts	Possible practical actions by the healthcare team
Fears	"Some people with Parkinson's experience fears related to their future with this disease. If this is something that happens to you sometimes, talking about such fears might offer valuable opportunities to spot and dispel misunderstandings, strengthen coping mechanisms, and plan ahead. What do you think?"	Listen to the patients' fears with empathy; normalize fears; think collaboratively with the healthcare team, the patient and their family about which coping mechanisms and resources could help assuage them; refer to psychotherapeutic support (e.g., dignity therapy, logotherapy)
Fear of disability	"When you think about the future, what possible losses worry you most, for instance, mobility, self-care, communication, or something else?"	Explain that the trajectories of Parkinson's disease are heterogeneous and shaped by multiple factors; focus on what can be planned and supported (e.g., function, safety, adaptive strategies) rather than predicting a single pathway; plan for function-focused goals; align rehab and safety planning with priorities; engage in advance care planning
Fear of shame related to intimate dependency	"Some people talk about feeling shame, especially when they think about being dependent on others for things like getting washed and dressed or going to the toilet. Are there aspects of support that make you feel embarrassed? What could make them more acceptable?"	Validate and normalize shame as a common response to loss of privacy; explore specific boundaries and preferences (e.g., same-gender caregiver, privacy measures, language used, timing); anticipate needs and plan practical supports (assistive devices, home care, continence and skin care plans); offer reframing focused on personhood and dignity without minimizing distress; consider referral to counseling when shame is intense or contributes to avoidance, depression, or social withdrawal.
Fear of being a burden to families/loved ones	"Some people worry about being a burden to their love ones. Have you ever felt like that?" What worries you most about how your illness affects the people close to you?"<br>"Have you ever talked to them about your fear of being a burden?"	Elicit relational priorities; assess carer needs, burden, and concerns; facilitate patient-preferred family conversations about shared hopes, limits, and supports; negotiate care plans; connect families to community resources. When appropriate, and with permission, use gentle perspective-taking to explore reciprocity and meaning in caregiving (e.g., "If you're comfortable, can we try a perspective shift? Imagine you were not the one with Parkinson's, but someone you love was. What would caring for them mean to you?")
Fear of pain and discomfort / symptom distress	"Are you afraid of having pain or other symptoms?" (If yes) "Which are most frightening?"<br>"Have you experienced those symptoms before because of Parkinson's or other illnesses, or are some of them things that you imagine could happen to you?"<br>"Have you witnessed someone having these symptoms?"	Prioritize symptom assessment and management plans; provide anticipatory guidance about possible end-of-life symptoms and available relief options; when symptoms are complex or refractory, consider specialty palliative/neuropalliative care consultation, including discussion of palliative sedation when clinically and ethically indicated.
Fear of abandonment / helplessness	"Who do you count on day-to-day?" "Have you ever been afraid of being abandoned or helpless?"	Map support network; plan contingencies; connect to community resources
Coping	"What things have given you strength to cope with Parkinson's?"	Elicit, validate, and reinforce coping resources (relational, practical, existential/spiritual) as part of longitudinal care planning; integrate

		coping goals into the care plan (e.g., maintaining meaningful roles/activities, supporting relational connection, addressing spiritual needs); coordinate interdisciplinary supports (e.g., psychology/psychiatry, social work, rehabilitation, chaplaincy) and revisit coping strategies as functional abilities change; identify lack of coping resources as potential red flag for complicated distress and refer when appropriate
Feeling well cared for	"What things help you feel well cared for?"<br>"What would you wish clinicians and your family would do or avoid?"	Translate the patient's description of "being well cared for" into observable care behaviors (e.g., continuity, kindness, respect, honest communication, attention to dignity); document these preferences and share them across team members; involve family/caregivers as appropriate while preserving the patient's voice.
Place of death	"In your last days of life, if comfort became hard to manage at home, would you wish to stay at home at all costs or would you be okay to go to a hospital or a nursing home?"	Clarify trade-offs; plan escalation pathways; align place-of-care with goals
Characteristics of healthcare professionals	"People value different things with regards to the healthcare professionals caring for them. What are your most important expectations concerning the attitudes and behaviors of healthcare professionals caring for you?"<br>"How much detail do you want about what may happen later, and how should we talk about it?"	Translate relational needs into care behaviors; align team approach; tailor communication; document preferences
Connection with families and loved ones	"How would you describe your relationship with your family and loved ones right now?"<br>"What would you hope from them in your last days of life?"	Explore relational goals and tensions (closeness vs protecting loved ones from distress); facilitate family conversations when desired by the patient (e.g., family meeting focused on hopes, worries, roles, and support needs); encourage anticipatory planning for practical caregiving arrangements while attending to emotional and relational needs; consider referral to counseling, caregiver support, or spiritual care when appropriate.
Finding opportunities for joy	"What brings you joy?"<br>"Are there special foods, music, or other activities that bring you joy?"<br>"What would you hope to keep doing as long as possible?"<br>"Are there specific people whose presence would bring you peace or joy? If so, how would you like that contact to happen (in person, phone, video), and when?"	Identify joyful activities and explore their feasibility; integrate into care goals; ask for input from occupational therapy; identify key people and relational goals; explore feasibility and preferences for contact/visits (in person or remote); address practical barriers (transportation, scheduling, hospital/long-term care visitation policies) with social work; plan "meaningful moments" (brief visits, shared rituals, music, photos) while optimizing comfort (fatigue, pain, anxiety) so encounters are possible; document joy and relational priorities and communicate them across the team
Religiosity/spirituality as coping and meaning	"Are faith or spiritual practices important to you as you think about the future? How can we support that?"	Spiritual assessment; chaplaincy/referral; integrate rituals/preferences
